# Mango Peel Pectin by Microwave-Assisted Extraction and Its Use as Fat Replacement in Dried Chinese Sausage

**DOI:** 10.3390/foods9040450

**Published:** 2020-04-07

**Authors:** Malaiporn Wongkaew, Sarana Rose Sommano, Tibet Tangpao, Pornchai Rachtanapun, Kittisak Jantanasakulwong

**Affiliations:** 1 Major of Biotechnology, Graduate School, Chiang Mai University, Chiang Mai 50200, Thailand; malaiporn@rmutl.ac.th; 2 Programme of Food Production and Innovation, Faculty of Integrated of Science and Technology, Rajamangala University of Technology Lanna, Chiang Mai 50300, Thailand; 3 Plant Bioactive Compound Laboratory, Faculty of Agriculture, Chiang Mai University, Chiang Mai 50200, Thailand; gr.zhk.88@gmail.com; 4 Department of Plant and Soil Sciences, Faculty of Agriculture, Chiang Mai University, Chiang Mai 50200, Thailand; 5 Cluster of Agro Bio-Circular-Green Industry (Agro BCG), Chiang Mai University, Chiang Mai 50200, Thailand; pornchai.r@cmu.ac.th (P.R.); antanasakulwong.k@gmail.com (K.J.); 6 School of Agro-Industry, Faculty of Agro-Industry, Chiang Mai University, Chiang Mai 50200, Thailand

**Keywords:** dried Chinese sausage, fat replacement, mango peel pectin, microwave-assisted extraction technique

## Abstract

In this research, low-fat dried Chinese sausage was formulated with mango peel pectin (MPP; 0%, 5%, 10%, and 15% (*w/w*)) extracted by microwave assisted extraction (MAE). The extractable yield of pectin attained from peel of Nam Dok Mai variety was achieved at 13.85% using 700-watt power. The extracted MPP were of high equivalent weight (1485.78 mg/mol), degree esterification (77.19%) and methoxyl content (19.33%) with a structure of greater porosity as compared to that of the conventional method. Spectrum scans by Fourier transform infrared spectrophotometer (FT-IR) indicated that the extracted MPP gave similar wave number profiles as the commercial pectin. Quality attributes of the Chinese sausages were assessed and compared with the control formula (CTRL). At higher concentrations of MPP, the intensity of redness and yellowness in sausage increased. The texture profile of the sausage illustrated that only the hardness value was comparable with the CTRL, while springiness, cohesiveness, gumminess and chewiness were statistically lower (*p* < 0.05). Furthermore, the sensory evaluation by experienced panellists (*n* = 12) indicated that 5% MPP similarly represented overall acceptability with the CTRL. Consequently, MPP can be effectively incorporated in the formula at low level to replace fat in Chinese sausage, allowing colour improvement and production of a healthier option.

## 1. Introduction

Fear of noncommunicable diseases (NCDs) has influenced the awareness of naturally functional ingredients in the human diet [1]. Additionally, this trend has motivated concerns of animal fat consumption, which encourages the novel formulation of products with reduced fat content [2]. Processed meats are usually products of high fat content, providing that fat could significantly improve texture, flavour, mouthfeel and perceived juiciness [3,4]. However, adding fat replacer into the product could adversely affect texture and their original sensory properties [5,6,7]. Moreover, excessive decrease in fat content can considerably alter the structural characteristics of meat products [8]. Chinese or Cantonese-style sausage, also called Kunchiang in Thai, is one of the preserved meat products of East Asian culinary heritage. The main ingredients are meats (pork or chicken) mixed with a high content of pork fat [9]. Attempts have been made to partly decrease or absolutely remove fat from Chinese sausage [10,11]. One option is by integrating functional ingredients, such as rice starch, gum and pectin, to replace the sum amount of lipid ingredient [12]. 

Dietary fibre is a carbohydrate polymer with more than 10 monomeric units, making it difficult to be hydrolysed by endogenous enzymes in the human small intestine [13,14]. The fibre can be classified into two groups videlicet, insoluble (cellulose and hemicellulose) and soluble (pectin, galactomannan, inulin and gum), depending on its solubility in aqueous solution [15]. Additionally, pectin is generally required as a food ingredient for the functional food industry [16]. Extractable pectin is utilised as a food additive that is promoted in the processes of gelling, stabilising and thickening [17]. Méndez-Zamora et al. [18] claimed that fat could be replaced with pectin and inulin in frankfurter sausages to produce healthy and functional products. The supplementation could also maintain the physical properties of meat product [19,20]. 

Mango peel is a potential source of dietary fibre with 5%–11% pectin depending on the extraction methods and also of fruit varieties [21,22,23]. Moreover, it comprises considerable various classes of polyphenols, carotenoids and vitamins with excellent antioxidative and functional properties [24,25], thus making this byproduct a promising target for commercial valorisation [26,27]. 

To recover pectin from plant resources, microwave-assisted extraction (MAE) is more effective for the extraction of high-quality pectin, compared with conventional heating techniques [28,29,30,31]. Such a technique has been adopted with pectin-rich biomasses such as banana peels [32], mango peels [22,33,34], pumpkin [35], and orange peels [36]. For Thai ‘Sampee’ mango variety, Sommano et al. [22,34] reported the improved recovering yield of mango peel pectin (MPP) by moderate microwave radiation and the process could also preserve bound phenolic content and antioxidant scavenging activities. Chaiwarit et al. [37] reported that MPP from ‘Nam Dok Mai’ variety could be a potential biopolymer for film formulation in drug delivery systems or edible film for food packaging. There is however, no research conducted on the functionality of MPP as a food additive in particular as fat replacer. With this rationale, the objectives of the present study were first to quantify the effect of MAE on functional properties of MPP of Nam Dok Mai variety and later to evaluate its potential to reduce fat content in Chinese sausage.

## 2. Materials and Methods 

### 2.1. Preparation of Mango Peel Powder 

Peel was removed from fully ripe mangoes Nam Dok Mai variety (L* = 50.90 ± 4.34, a* = 4.82 ± 2.35, b* = 16.59 ± 3.09; peel thickness = 138.76 ± 10.55 mm and percentage of peel to fruit weight = 5.31 ± 0.38%). The peels were cut into small pieces, washed with tap water, blanched with hot water at 95 °C for 10 min, drained and left cooled at room temperature, prior to drying at 60 ± 1 °C until the moisture content of 4%–6% was reached [38]. The dried peel was ground to a fine powder in a high-speed food processor and passed through a sieve, resulting in a final mass of particles smaller than 0.6 mm in diameter [39,40]. 

### 2.2. Extraction of Mango Peel Pectin Using Microwave-Assisted Technique

Twenty grams of mango peel powder was suspended in 600 mL of diluted acidic solution (distilled H_2_O adjusted to pH 1.5 with 2 M HCl) and soaked for 20 min at room temperature. The slurry was heated in a microwave oven (ME711K-XST, Thai Samsung electronics Co., Ltd., Bangkok, Thailand) with an output power of optimal condition (700 watts for 3 min) followed by recooling to room temperature [22]. The solution was filtered and pressed manually using a nylon cloth. The filtrates were centrifuged at 5000× *g* for 20 min to eliminate any remaining coarse particles. Pectin was precipitated from this clear supernatant by adding the same volumes of ethanol (95%); mixed and stored in a refrigerator at 4 °C for 30 min. The separation was achieved by vacuum filtration. The obtained pectin was dried in a hot air-oven at 40 °C until constant weight [41]. The yield (%) of pectin was calculated from the following equation [40];

(1) $$\mathrm{Yield}\;\left(\%\right)=\left(\frac{M_{0}}{M}\right)\times \;100$$

where M_0_ (g) = the weight of dried pectin and M (g) = the weight of dried mango peel powder.

### 2.3. Scanning Electron Microscope

Pectin powder was attached onto a specimen stub with a double-sided tape and sputter coated with gold [22,42]. The images were viewed at magnifications of × 100 and × 500 using SEM (JEOL JSM-5910, Japan Electron Optics Laboratory Co., Ltd., Tokyo, Japan) with an accelerating voltage of 10 kV. 

### 2.4. Fourier Transform Infrared Spectrophotometer (FT-IR)

FT-IR analysis was implemented using an infrared spectrometer (Nicolet, USA) equipped with an MCT Detector (mercury cadmium telluride). Each sample was scanned by placing the sample side down on the ATR diamond crystal and applying the pressure tower. The spectrum was verified in the transparent mode from 900 to 4000 cm^−1^, with a resolution of 4.0 cm^−1^ [22]. Each IR spectrum was improved for optical effects with the ATR correction algorithm (OMNIC software).

### 2.5. Mango Peel Pectin Characterisations 

The equivalent weight (Eq.W) was determined by the method of Ranganna [43]. Briefly, 0.5 g of dried pectin was dissolved in 100 mL of distilled water at 25 °C and stirred for 2 h until completely dissolved. One gram of sodium chloride was added and titrated with 0.1 M of sodium hydroxide (NaOH) using 5 drops of phenol red as an indicator. Eq.W was calculated using the following equation:

(2) $$\mathrm{Eq}.W=\frac{1000\;\times \;\mathrm{pectin}\;\mathrm{powder}\;(g)}{\mathrm{NaOH}\;\mathrm{concentration}\;\left(N\right)\;\times \;\mathrm{NaOH}\;\mathrm{volume}\;(\mathrm{mL})}$$

Methoxyl content (Mox) and degree of esterification (DE), the methods suggested in Ranganna [44] and Pinheiro et al. [45], were followed. Dried pectin (0.2 g) was stirred in CO_2_-free distilled water (20 mL) until fully dissolved. One gram of NaCl was added to the solution, prior to titrating with 0.1 N NaOH in the presence of phenolphthalein. The volume was recorded as the initial titre (V_1_). Then, 0.1 N NaOH solution (10 mL) was added to a neutralised polygalacturonic acid sample after the determination of the free carboxyl groups. The solution was mixed thoroughly until the colour of the solution became purple. A few drops of the indicator (0.25 N HCl) were added, and the mixture was titrated with 0.1 N NaOH until the colour turned from yellow to pink. The volume was noted as V_2_. The Mox and DE were then calculated using the following equations;

(3) $$\mathrm{Mox}=\;\frac{{(N)(V}_{2})(E)}{1000\;(S)}$$

(4) $$\mathrm{DE}=\;\frac{V_{2}\;\times \;100}{V_{1}+\;V_{2}}$$

where S = mass of dried pectin (g); N = NaOH concentration (N); V1 = volume of NaOH used (mL); V2 = volume of NaOH used (mL) and E = equivalent weight of methoxyl = 31

The water holding capacity (WHC), oil holding capacity (OHC) and swelling capacity (SWC) were evaluated following the method of Robertson et al. [46] with some modification. Phosphate buffer (1 M, pH 6.3, 25 mL) or commercial olive oil were added to 250 mg of dry sample, stirred thoroughly and left at room temperature for 1 h. The residue was weighed after centrifugation at 3000× *g* for 5 min. For SWC analysis, 0.1 g of sample was hydrated in 10 mL of distilled water in a calibrated cylinder (15 cm diameter) at room temperature. After equilibration for 18 h, the bed volume was documented. The WHC was expressed as amount of water (g) held per sample (g); the OHC was expressed as amount of oil (g) held per sample (g), while the SWC was expressed as mL/g of sample.

Colour analysis of MPP powder was measured according the method of Bolumar et al. [47] with some modification. The colour was assessed using a handheld colour spectrophotometer (NS800, 3nh, China). Before each set of measurements, the instrument was calibrated using a white ceramic tile. The measurement was with CIE Lab system; where L* denotes lightness on a 0 to 100 scale from black to white; a* denotes (+) red or (−) green and b* denotes (+) yellow or (−) blue.

### 2.6. Processing and Evaluation of Dried Chinese Sausage with Added Mango Peel Pectin

#### 2.6.1. Dried Chinese Sausage Formulation

Chinese sausage ingredients (CTRL) obtained from Chiang Mai Livestock Product Research and Development Centre consisted of pork, fat, sugar, sodium nitrite, sodium erythorbate and water at (%*w/w*) 60.0, 20.0, 12.0, 1.2, 0.1 and 6.7, respectively. Pork and fat were ground and then mixed with all ingredients. Pectin powder was added at a level of 5%, 10% and 15% (*w/w*) fat replacement. It was firstly dissolved with 2 g of clean water and then mixed with the prepared ingredients for 10 min with cutter mixer (QS600, Baicheng, China). After that, the ingredients were added in dried pork sausage casing and dried in a hot air-oven at 60 ± 5 °C for 48 h. The sausages were left to cool at room temperature, packed in vacuum nylon bag and stored at 4 ± 1 °C.

#### 2.6.2. Colour Evaluation

Chinese sausages added with 0%, 5%, 10% and 15% (*w*/*w*) of MPP were sliced into 10-mm thickness. The colour measurement was repeated 10 times at different parts of the sausage surface using a handheld colour spectrophotometer (NS800, 3nh, China). Before each set of measurements, the instrument was calibrated using a white ceramic tile. The measurement was with CIE Lab system; where L* denotes lightness on a 0 to 100 scale from black to white; a* denotes (+) red or (−) green; and b* denotes (+) yellow or (−) blue. To compare the overall colour changes between the MPP-supplemented Chinese sausage samples and the CTRL, the total colour differences (ΔE) between the samples (L*, a*, b*) and the CTRL (L_0_*, a_0_*, b_0_*) were calculated as presented below [47,48];

(5) $${\Delta E}_{\mathrm{ab}}=\sqrt{{{(L*\;-\;L}_{0}*)}^{2}{+(a*\;-\;a}_{0}{*)}^{2}{+(b*\;-\;b}_{0}{*)}^{2}\;}$$

#### 2.6.3. Texture Profile Analysis

The sausages sliced for colour measurement were also used for Texture Profile Analysis (TPA) using a TA-TX2 texture analyser (Stable Micro Systems Ltd., Godalming, UK), attached with a 50-kg load cell. A 50-mm diameter compression cylindrical aluminium probe was used to compress a cylindrical shape of the sausage, which was compressed twice to 30% of the original height of the sausage at a compression rate of 1.0 mm/s at room temperature. The TPA settings were as follows: pretest speed: 2.0 mm/s; test speed: 1.0 mm/s; post-test speed: 2.0 mm/s; target mode distance: 3.0 mm; trigger force: 5 g; trigger type: auto and data acquisition rate: 200 points per sec. (pps). The delay between the first and second compression was 5 sec. The TPA analysis was carried out at ambient temperature (25 °C), and the analysis was completely operated within 17 sec. Six measurements were assessed for each sample in the same lot. A force-time graph was generated and textural parameters, including hardness, cohesiveness, springiness, gumminess and chewiness, were calculated with software provided along with the instrument [49].

#### 2.6.4. Sensory Test

Sensory evaluation of the Chinese sausage products was operated following the modified procedures by Siddaiah et al. [50] using a panel of 12 individuals from Chiang Mai Livestock Product research and Development centre, who had been trained for the sensory assessment of Chinese sausage and with a minimum of 5-years-experience in meat processing. All the panels were assured that they understood the definitions of appearance, juiciness, springiness, firmness, colour and overall acceptability before the panel tested the Chinese sausage. Preparation of the meat products for testing occurred in a kitchen separated from the evaluation room, eliminating possible interference of fried odour. According to routine sensory evaluation of Chiang Mai Livestock Product and Development Centre, the sausage samples were sliced into 7-mm thickness and then fried with palm oil for 3 min in a low heat. Each panellist was given two pieces of each sample for evaluation on nine-point hedonic scale (1 = strongly dislike and 9 = strongly like).

### 2.7. Statistical Analysis

All experiments were operated with at least triplicate samples for each test. Data was analysed using one-way analysis of variance and Duncan’s test. Differences in values were considered significant when the p value was <0.05. All statistical analysis was performed using SPSS program (version 22.0, Armonk, NY, USA).

## 3. Results and Discussion

### 3.1. Scanning Electron Microscope

**Figure 1:**
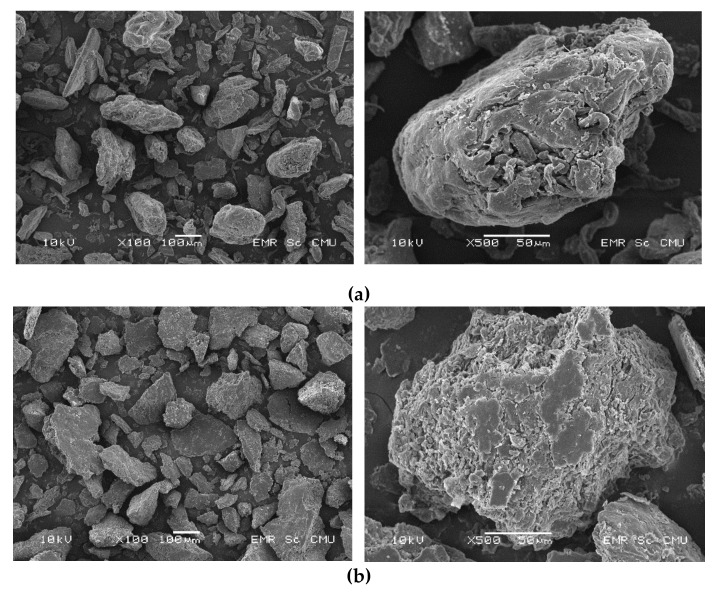
The SEM images of commercial pectin (citrus) (**a**) and pectin obtained using microwave-assisted extraction (MAE) from peel of Nam Dok Mai mango at 700 watts (**b**). The images were viewed at ×100 (left) and ×500 (right).

SEM was performed to characterise the surface of commercial citrus pectin (Figure 1a) and our MPP (Figure 1b) samples by visualising their structures and morphology. The images demonstrate that the pectin particles are of distinct shapes, Nam Dok Mai MPP illustrates pellets to bulky and rough particles, which differs greatly from the shape of the commercial pectin, which has a comparatively smooth surface. Nevertheless, the MPP particles extracted using MAE 700 watts are crumblier in shape and with more porous surfaces. Begum et al. [51] reported that the dehydrated pectin obtained from jackfruit freeze-dried and spray-dried had high solubility due to their high porosities, smaller particle size and higher surface area. Thus, pectin particles with more porous structures usually have a better solubility than particles with the rigid structure and lower porosity, thereby increasing solution viscosity [52]. The porous quantity of pectin was correlated with water holding capacity and led to the low hardness property of low-fat frankfurter sausage [18]. The dietary ingredients influence the high binding ability and water holding capacity of meat product [6]. According to dielectric mode of action, microwave is in fact more efficient than other extraction methods due to the strong formation of vapour in polar substances created by the electromagnetic field [53]. Heat vapour modifies the cell wall matrix and leads to the severing of parenchymal cells, which rapidly and extensively opens the skin tissues, thus increasing the interaction between the extracting agent and the plant material during the extraction process [31]. Furthermore, the images of the MPP (Figure 1b) suggest a rough, ruptured and wrinkled surface, which could be due to the sudden increase of temperature in the MAE process. Similarly, Liew et al. [54] reported that the coarse surface of the extracted pectin using MAE could be due to the rapid rise in temperature. Sources of raw materials as well as modes of extraction could largely influence morphology of the resulted pectin [28]. Regarding commercial citrus pectin morphology, the surface showed multilaminate structures and was fluffy with a smooth surface [55], which was considerably different from the MPP surface. From their high porosity, MPP is appropriate for fat replacer in Chinese sausage.

### 3.2. FT-IR Analysis of MPP

**Figure 2:**
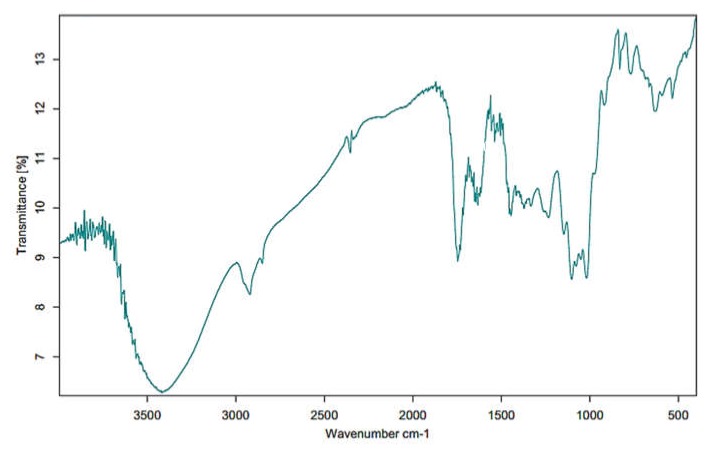
The FT-IR spectra of pectin extracted from Nam Dok Mai mango peel using MAE at 700 watts, from 900 to 4000 cm^−1^ (x axis). %T is the percentage of transmittance (y axis).

The FT-IR analysis is generally used to evaluate the conformation of pectin bands in the standard region usually between 1000 and 2000 cm^−1^ for the major chemical and functional groups [56]. Figure 2 illustrates the FT-IR region ranging from 900 to 4000 cm^−1^ of MPP. These demonstrate the similarities of the transmittance (%T) patterns in pectin extracted from different source materials. An individual peak at around 3400 cm^−1^ is likely due to the stretching of the hydroxyl groups, whereas a small peak at around 3000 cm^−1^ indicates C–H stretching of the CH_2_ groups [42]. The strong absorption at 1730–1760 cm^−1^ represents the characteristic of esterified pectin, arising from the ester carbonyl-stretching band, and peaks at 1600–1630 cm^−1^ and 1400–1450 cm^−1^ are due to the antisymmetric and symmetric stretching frequencies of the ionic carboxyl groups [57]. The region at wavenumbers between 1500 and 1800 cm^−1^ is associated with the assessment of the degree of methylation [58]. Thus, the peak at around 1730 cm^−1^ in the pectin spectra corresponds to a higher DE value [59]. The region between 950 and 1200 cm^−1^ is accordingly referred to as the ‘fingerprint’ for carbohydrates, especially sugar composition [60]. The intense peaks relate to the characteristics of pectin polysaccharides (polygalacturonic acid) performed at 962, 1024, 1099, 1156 and 1223 cm^−1^, which are assigned to C–O bending, C–C stretching, C–O stretching, C–H stretching and C–O stretching, respectively [59]. FT-IR analysis verified that the extracted constituent was pectin. Similar band patterns were detected in pectin extracted from Sam-pee mango [22], banana peel [61] and lime peel [59].

### 3.3. Characterisation of Mango Peel Pectin

Table 1 illustrates the characterisation of Nam Dok Mai MPP extracted by conventional and MAE techniques. The average yield of MPP extraction operated by conventional heating was approximately 0.80% [22], which was dramatically low when compared to the quantity of pectin extracted by MAE at 700 watts (13.85%). Microwave extraction gave better pectin recovery when compared to conventional extraction. Similarly, MAE was reported to be an applicable mode of extraction for high yield pectin recovery in grapefruit (27.81%) and navel orange peel (18.13%) [39,62]. Microwave heating is indeed more efficient than other extraction methods due to the intense formation of vapour in polar substances generated by the electromagnetic field [53]. Heat vapour modifies the cell wall matrix and leads to the severing of parenchymal cells, which rapidly and extensively break down cell membrane, thus increasing the interaction between the extracting agent and the plant material during the extraction process [31]. In addition, microwave energy also results in the inactivation of the pectinase [53].

Colour of pectin is an essential parameter as it influences the appearance of the formulated products. The colours of MPP obtained from conventional and MAE technique are shown in Table 1. L*, a* and b* values of MPP extracted by both techniques are not significantly different (*p* > 0.05). Comparing the lightness (L*), our extracted MPPs were slightly darker than that of commercial citrus peel pectin extracted using the same extraction method [59]. To this end, pigmentation of the biomass could play an important role as the pigments cannot be removed by extraction steps. Moreover, nonenzymatic browning reactions, i.e., Maillard reaction and caramelisation, are also influenced by heating and might be of great contribution to the pectin colour [63]. In addition, high pigmented pectin may be a result of bound polyphenols [64] or other water-soluble pigments. Different extraction conditions (time and temperature) could also affect pectin colour [65].

Equivalent weight (Eq.W) of pectin is an indicator of gel-forming ability. The greater the Eq.W, the higher the gel-forming ability achieved [66]. The Eq.W of the MPP was about 1400 mg/mol, which was two-fold higher than that of the conventional extraction. The values are comparable with citrus pectin illustrated ranges of Eq.W between 635.63 to 2219.39 mg/mol depending on the extracting methods [59]. Pectin recovered by MAE seems to give a higher Eq.W than that of the conventional heating. The lower Eq.W could be due to higher partial degradation of pectin, thus the variation of Eq.W value depended upon the amount of free acid [67]. Consequently, it can be indicated that the heating of microwave does less damage to the pectin structure than that of the conventional method.

Methoxyl (Mox) content is an essential indicator of pectin setting time, their sensitivity to polyvalent cations and their beneficial properties in the preparation of low solid gels, films and fibres [68]. Moreover, Mox also represents the pectin distribution ability in water and gel ability [69,70]. Pectin extracted by MAE at 700 watts gave 19.33% Mox, which was significantly higher than that of the conventional extraction (13.90%) [22]. Commercially, a high methoxyl pectin (generally at 8%–11% Mox) can form gels at a high sugar content (>65% sugar), while a low methoxyl pectin with less than 7% Mox can form gels at a lower sugar content [71]. In this study, MPP was classified as high methyl pectin due to the higher Mox (>8%); therefore, it needs a very high sugar content (>65%) to suit high methoxyl pectins [59].

DE is a significant molecular index for pectin classification that defines the extent to which carboxyl groups in pectin molecules exist as the methyl ester [72]. The DE value of pectin extracted by MAE from Nam Dok Mai mango peel is 77.19%, which is higher than using a conventional heating method (68.90%). In a similar study, MAE of pectin from lime albedo, pulp and flavedo produced higher DE values than those of conventionally extracted pectin [28]. According to the DE values, MPP extracted by MAE can be classified as high methoxyl pectin as DE > 50% [36]. In addition, the pectin would have a rapid-set gel formation at DE > 72% [69].

Swelling capacity (SWC), water holding capacity (WHC) and oil holding capacity (OHC) of MPP extracted using MAE 700 watts are illustrated in Table 1. In comparison with the conventional MPP, SWC, WHC and OHC increased but were not significantly different from the MAE technique. It was advised this was due to the lower degree of esterification, the greater WHC and other physical properties the pectin demonstrated [73]. SWC elucidates how much the fibre matrix swells when water is absorbed. The high SWC is correlated with the amount of soluble dietary fibre, especially pectin [74]. The SWC value acquired for MPP (24.16 mL/g sample) is greater than those obtained for other fruit fibre, including those from passion fruit pulp, peel and seeds (7.2 mL/g sample) [75] or cocoa pod husks (5.81 mL/g sample) [76]. This value defines the structural characteristics and chemical composition of the fibre that play an important role in the kinetics of water uptake [77].

WHC is the ability of a moist material to hold water when subjected to an external centrifugal gravity force or compression. The value consists of the sum of linked water, hydrodynamic water and physically trapped water, the latter of which contributes most to this capacity [78,79]. WHC of MPP was 9.60 g water/g sample. High WHC value demonstrates that MPP has potential applications in products that require high viscosity and texture improvement, such as cooked meat or bakery [74,80]. To this, Boulos et al. [81] explained that water molecules either as free or bound forms react with carbohydrates in the association of heat. The linear molecules such as amylose and amylopectin are realigned into an immobile monolayer to form a precipitate or a gel, a phenomenon known as retrogradation. This therefore increases the viscosity ability of the carbohydrate.

OHC is a physical property associated with the chemical structure of plant polysaccharides and depends on surface properties, overall charge density, thickness and the hydrophobic nature of the fibre particle [82]. Our MPP showed a considerably lower OHC (0.81 g oil/g sample) than other fruit and vegetable-derived fibres, such as passion fruit albedo, 2.03 g oil/g sample [74], pomegranate bagasse, 5.9 g oil/g sample [83] or ripe kiwi 6.00 g oil/g sample [84]. As a result of its low OHC, the extractable MPP has potential ingredients for fried products since it would not provide a greasy sensation [74,85].

### 3.4. Physical Quality Assessments of Formulated Dried Chinese Sausage

#### 3.4.1. Colour

Lightness (L*), redness (a*), and yellowness (b*) are considered the most informative parameters for quality assessment of product [86]. Surface colour of the dried sausage supplemented with MPP is illustrated in Table 2. From the result, it can be described that the higher concentration of the MPP added to the sausage, the lower the value of lightness. Our result also illustrates that the redness and yellowness of the sausage increases in all formulated products, and the colour intensity is higher with the increasing concentrations of the MPP. The result is correspondent with the report of Sarıçoban et al. [87] who found that the carotenoids as a food additive improved the redness in meat batters. Compared with the CTRL (0% pectin), ΔE values of the formulated products were significantly distinct from the CTRL (*p* < 0.05) ranging from 9.91–5.55 from the highest to the lowest concentrations, respectively. For this, it is possible that MPP could increase product colour intensity. The results corresponded well with that of Almeidat et al. [88], who advised that fat replacement with a high amount of amorphous cellulose (75% and 100%) in emulsified cooked sausage reduced the surface lightness of the product. Regardless of the product mouthfeel, it might be a promising option to adjust the colour of sausage by adding differently treated MPP [89]. The other textural enhancements such as protein isolate and starch however, affected colour of the meat product differently. Moreover, the protein isolate from pea can enhance cod sausage colour towards higher b*(yellowness) depending on ingredient mixtures and their concentrations [5]. Likewise, the addition of quinoa flour in frankfurter sausage significantly increased colour intensity of the product [90]. On the contrary, the resistance starch addition had no influence on the sausage colour [91]. In this study, addition of MPP had considerably altered the colour of Chinese sausage due to the bioactive compounds, especially carotenoid consisting in ripe mango peel [34,92,93]. This is quite beneficial for the use of dietary fibre of this kind as a functional ingredient.

#### 3.4.2. Texture

**Figure 3:**
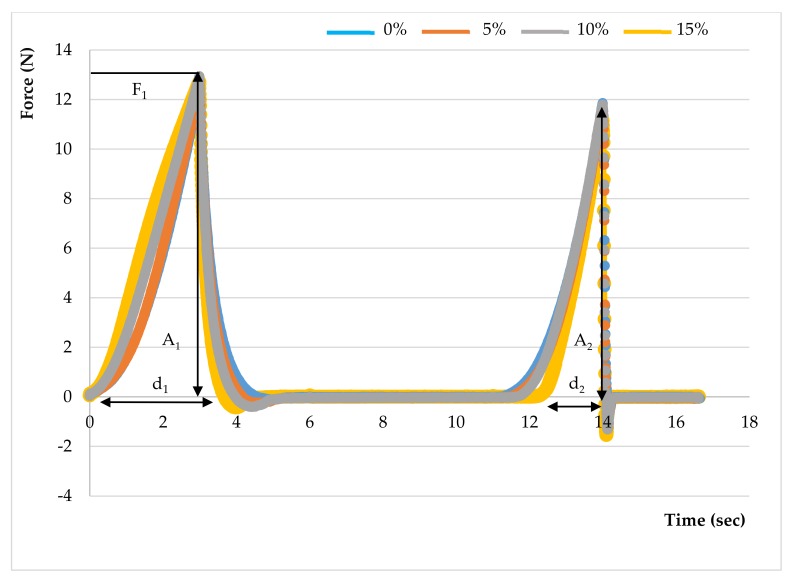
Texture profile of dried Chinese sausage with added mango peel pectin at different levels. Where; Hardness: F_1_; Cohesiveness: A_2_/A_1_; Springiness: d_2_/d_1_; Gumminess: Hardness × Cohesiveness and Chewiness: Gumminess × Springiness.

The force-deformation curves of the formulated samples are represented in Figure 3. The textural behaviour of sausages with MPP concentrations of 0%, 5%, 10% and 15% (*w/w*) are shown in Table 3. The hardness is the maximum peak force (F_1_) during the first compression cycle required to compress a food between the molar teeth [94]. From the result, the hardness of all formulated samples was not significantly different (*p* > 0.05), whereas springiness, cohesiveness, gumminess and chewiness were lower in treatments with the pectin fibres (*p* < 0.05). The CTRL had the highest hardness value of 15.87 N followed by adding 5%, 10% and 15% (*w/w*) pectin powder with the values of 13.15, 12.89 and 12.70 N, respectively. Cierach et al. [95] described that the hardness in the sausages was related to their fat content. The higher MPP added to the sausage formula, the smaller the slope of the first peak obtained (hardness) (Figure 3). These differences in hardness profiles could be due to the binding ability and water holding capacity of fat and MPP mixture [6]. It could be obviously seen that the texture of sausages with addition of MPP were softer. This could be in association with gel strength of pectin quantity under compression [96]. According to Campagnol et al. [97], the hardness of fermented sausages added with amorphous cellulose as fat replacement at levels of 50%, 75% and 100% (*w/w*) was not significantly different from the control.

The springiness is a textural parameter, which is correlated with elasticity of the sample. For texture profile analysis, springiness is associated with reversible ability of food after the end of first bite and the begin of the second bite. If springiness is high, it requires more mastication energy in the mouth [98]. The springiness values of four sausage samples are also represented in Table 3. There was a significant difference in the springiness values of all treatments of the sausages (*p* < 0.05). The sausage added with 15% (*w/w*) MPP showed the lowest springiness value compared with other samples. The higher concentration of MPP added, the lower springiness value obtained. Zapata and Pava [90] reported that quinoa flour supplementation had no significant influence on the springiness of frankfurter sausage. Whereas the higher MPP concentration negatively affected the springiness of Chinese sausage samples because of the gelling characteristic [12].

The cohesiveness (consistency) indicates the strength of internal bonds making up the body of food and the degree to which a food can be deformed before it ruptures (breaks) [99]. Cohesiveness is defined as the ratio of the positive force area during the second compression to that of the first compression. It also indicates the ability of the product to hold together [96]. The cohesiveness values of the sausage samples were in the ranges of 0.28 to 0.50. The highest and lowest values obtained were for 0% and 15% (*w/w*) of the pectin supplementation, respectively. Garcia-Santos et al. [91] revealed that the sausage with the addition of resistant starch had a low value of cohesiveness (0.50–0.70). Choe et al. [100] also reported the cohesiveness values of sausages supplemented with wheat fibre for the reduction of fat ranged from 0.27 to 0.34. Troutt et al. [101] found that the addition of three-ingredient combinations of Polydextrose^®^, potato starch and either sugar beet, oat or pea fibre reduced cohesiveness of beef patties. While quinoa flour had no noticeably effect on the cohesiveness value of frankfurter sausage [90]. The more supplementation of MPP in the Chinese sausage, the lower the value of cohesiveness (*p* < 0.05) because gelling was formed at higher concentration.

Gumminess is defined as the product of hardness and cohesiveness. It is a characteristic of semisolid foods with a low degree of hardness and high degree of cohesiveness. From Table 3, it can be seen that higher amount of MPP resulted in the lower values of gumminess; however, the values of Chinese sausages supplemented with MPP were not significantly distinguished (*p* > 0.05). The higher gumminess has also ascended from the higher hardness value [98]. Regarding research by Cardoso et al. [102], the gumminess value of cod frankfurter sausage remarkably increased (*p* < 0.05) with pea protein supplementation. Méndez-Zamora et al. [18] also found the gumminess of frankfurter sausages replacing fat with inulin and pectin was lower when a higher amount of pectin was added. In this research, Chinese sausage samples supplemented with MPP represented both visco-elastic and gumminess behaviour from the pectin attribute.

Chewiness is a measure of energy required to masticate the food and is normally reported for solid foods. It is defined as the product of gumminess and springiness [96]. The chewiness value of four Chinese sausage samples varied from 2.18 to 7.94 N. There was a significant difference in the value of all sausage treatments (*p* < 0.05). Similarly, higher amount of MPP powder supplemented in the sausage also led to a lower value of chewiness. Feng et al. [103] found statistical differences of gumminess between low-fat Chinese sausages supplemented with Mesona Blumes gum or rice starch mixed gels (*p* < 0.05). Cardoso et al. [102] reported that the chewiness value of cod sausage statistically increased (*p* < 0.05) with pea protein and carrageenan integration. The results could be due to the absence of a water content adjustment, causing moisture to decrease while protein and carbohydrate contents increased. On the other hand, the chewiness value of the Chinese sausages with MPP additive noticeably descended with the higher pectin levels. Because of the presence of high water content in the sausages, it could enhance swelling and gelling properties of the pectin.

Regarding all texture results, MPP influences on texture attributes of Chinese sausage due to their functional characteristics of pectin are used as gelling and texture modified agent in meat products [12]. Consequently, Chinese sausage supplemented with a low amount of the pectin has considerably similar texture properties of the conventional sausage.

#### 3.4.3. Sensory Evaluation

Sensory evaluation can assist food scientists in instructively gaining a distinct understanding of the consequences of reformulation low-fat meat processes. Table 4 represents the acceptance of the sensory attributes of Chinese sausages added with MPP. Each sample was evaluated by 12 trained panels (sex: 6 females, 6 males; age = 25–40 years). The addition of pectin in the levels of 5% and 10% (*w/w*) slightly influenced (*p* > 0.05) the sensory attributes compared with the CTRL. However, the maximum pectin amount (15%(*w/w*)) shows the least acceptance scores in all parameters. Regarding overall acceptability, five percentage of the pectin was the favourite treatment because of its juiciness and appearance. With similar texture attributes (Table 3), the low pectin level added in sausage was more accepted than higher levels. Méndez-Zamora et al. [18] reported that higher levels of pectin added in low-fat frankfurter sausage affected the flavour and odour. Rahman et al. [104] reported that fish sausages with higher starch content had given higher sensorial hardness. Lin and Huang [105] revealed that the konjac or gellan gum additive could improve the firmness of low-fat frankfurter sausage owing to the reduction of fat. Feng et al. [103] found the Mesona Blumes gum or rice starch mixed gels still exhibited the properties of juiciness, facilitating a better overall acceptability of the low-fat Chinese sausage. From sensory evaluation of low-fat Chinese sausage added MPP results, it can be primarily summarised that MPP at high concentrations had dramatically influenced Chinese sausage sensory attributes after sample preparation by pan-frying.

## 4. Conclusions

The microwave-assisted extraction technique evaluated in this study has successfully proven to be a complementary method for the extraction of mango pectin. Consequently, we achieved a significantly greater pectin yield from peel of Nam Dok Mai mango with the MAE 700 watts. The equivalent weight, methoxyl content and degree of esterification of MPP processed higher than that of the conventional method. The substitution of 5% pectin to fat content in the Chinese sausage could enhance colour and conserve the physical qualities as well as sensory attribute. In conclusion, MPP can be utilised in the low-fat Chinese sausage formula as a novel fat replacer.

## Figures and Tables

**Table 1:** Qualities and functionalities of mango peel pectin extracted by conventional and MAE techniques.

Extraction Techniques	Qualities of Pectin							Functionalities of Pectin		
	Pectin Yield (%)	L*	a*	b*	Eq.W (mg/mol)	Mox (%)	DE (%)	SWC (mL/g sample)	WHC (g water/g sample)	OHC (g oil/g sample)
MAE 700	13.85 ± 0.51	36.33 ± 1.11	5.25 ± 1.05	11.26 ± 2.13	1485.78 ± 0.74	19.33 ± 0.04	77.19 ± 0.72	24.16 ± 0.22	9.60 ± 0.46	0.81 ± 0.04
Conventional	0.80 ± 0.06 [34]	36.88 ± 0.18	5.00 ± 0.20	11.39 ± 0.62	657.89 ± 47.33 [34]	13.90 ± 2.10 [34]	68.90 ± 3.70 [34]	25.50 ± 0.61	11.10 ± 0.23	1.04 ± 0.05
ANOVA Test		Ns	Ns	ns				ns	ns	ns

Data are expressed as mean ± standard deviation, n = 3; MAE 700 = microwave-assisted extraction at 700 watts; qualities and functionalities of mango peel pectin was according to our observation and those reported by Sommano et al. [34]. Eq.W = equivalent weight; Mox = methoxyl content; DE = degree of esterification; SWC = swelling capacity; WHC = water holding capacity and OHC = oil holding capacity.

**Table 2:** Colour of dried Chinese sausage with added mango peel pectin at different levels.

Percentage of Pectin	L*	a*	b*	ΔE	
0 (CTRL)	53.60 ± 7.44 ^a^	6.69 ± 2.40 ^c^	7.27 ± 1.32 ^c^	-	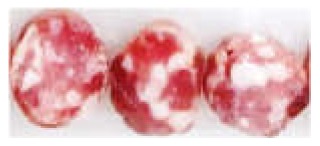
5	52.88 ± 2.87 ^a^	9.36 ± 0.80 ^b^	11.25 ± 0.62 ^b^	5.55 ± 1.02 ^a^	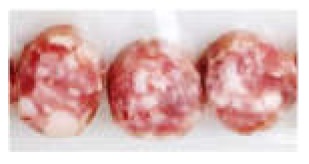
10	55.42 ± 1.82 ^a^	10.46 ± 0.69 ^ab^	13.31 ± 0.85 ^a^	7.59 ± 0.74 ^b^	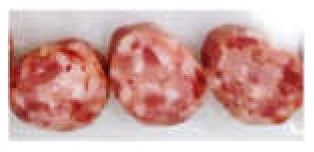
15	50.37 ± 3.81 ^a^	12.16 ± 1.17 ^a^	14.14 ± 0.74 ^a^	9.91 ± 2.12 ^c^	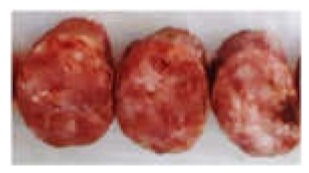

Data are expressed as mean ± standard deviation, *n* = 10; mean values with the same lowercase superscript letter of the same colour value are not significantly different (*p* > 0.05).

**Table 3:** Texture profile analysis of dried Chinese sausage with added mango peel pectin at different levels.

Texture Characteristics	Percentage of Mango Peel Pectin			
	0 (CTRL)	5	10	15
Hardness (N)	15.87 ± 3.45 ^a^	13.15 ± 0.66 ^a^	12.89 ± 2.26 ^a^	12.70 ± 1.48 ^a^
Springiness (mm)	1.00 ± 0.01 ^a^	0.84 ± 0.07 ^b^	0.78 ± 0.06 ^b^	0.61 ± 0.04 ^c^
Cohesiveness	0.50 ± 0.01 ^a^	0.39 ± 0.06 ^b^	0.37 ± 0.03 ^b^	0.28 ± 0.02 ^c^
Gumminess (N)	7.92 ± 1.78 ^a^	5.15 ± 0.99 ^b^	4.78 ± 0.99 ^b^	3.54 ± 0.52 ^b^
Chewiness (N.mm)	7.94 ± 1.80 ^a^	4.40 ± 1.15 ^b^	3.78 ± 1.04 ^bc^	2.18 ± 0.41 ^c^

Data are expressed as mean ± standard deviation, *n* = 6; mean values with the same lowercase superscript letter of the same texture characteristic are not significantly different (*p* > 0.05).

**Table 4:** Sensory analysis of dried Chinese sausage with added mango peel pectin at different levels with 9-points hedonic scale scoring.

Parameters	Percentage of Mango Peel Pectin (*w*/*w*)			
	0%	5%	10%	15%
Appearance	7.42 ± 2.15 ^a^	7.08 ± 1.08 ^a^	5.83 ± 1.53 ^ab^	4.92 ± 1.83 ^b^
Juiciness	8.33 ± 0.89 ^a^	6.92 ± 1.16 ^a^	6.83 ± 0.94 ^a^	5.42 ± 1.56 ^a^
Springiness	6.75 ± 1.66 ^a^	6.75 ± 1.82 ^a^	6.17 ± 1.53 ^ab^	4.00 ± 2.45 ^b^
Firmness	6.08 ± 2.02 ^a^	6.17 ± 1.70 ^a^	5.92 ± 1.56 ^a^	4.00 ± 2.00 ^b^
Colour	5.58 ± 2.23 ^a^	6.00 ± 2.00 ^b^	5.83 ± 1.70 ^b^	4.08 ± 2.68 ^c^
Overall acceptability	6.58 ± 1.68 ^a^	6.58 ± 1.56 ^a^	6.00 ± 1.41 ^a^	3.08 ± 1.88 ^b^
Prepared sausage for sensory	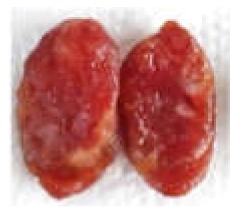	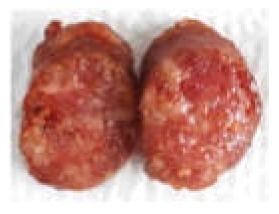	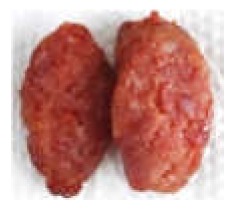	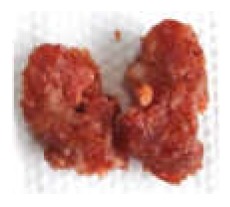

Data are expressed as mean ± standard deviation, *n* = 12; mean values with the same lowercase superscript letter of the same parameter are not significantly different (*p* > 0.05).
